# Comorbidities in people with hand OA and their associations with pain severity and sensitization: Data from the longitudinal Nor-Hand study

**DOI:** 10.1016/j.ocarto.2023.100367

**Published:** 2023-05-05

**Authors:** Elisabeth Mulrooney, Tuhina Neogi, Hanne Dagfinrud, Hilde Berner Hammer, Pernille Steen Pettersen, Marthe Gløersen, Tore K. Kvien, Karin Magnusson, Ida K. Haugen

**Affiliations:** a Center for Treatment of Rheumatic and Musculoskeletal Diseases (REMEDY), Diakonhjemmet Hospital, Oslo, Norway; b Faculty of Medicine, University of Oslo, Oslo, Norway; c Section of Rheumatology, Boston University School of Medicine, Boston, MA, United States; d Lund University, Faculty of Medicine, Department of Clinical Sciences Lund, Orthopaedics, Clinical Epidemiology Unit, Lund, Sweden

**Keywords:** Osteoarthritis, Hand osteoarthritis, Pain, Pain sensitization, Comorbidity

## Abstract

**Objective:**

To determine whether the comorbidity burden and co-existing comorbidities are cross-sectionally and/or longitudinally associated with pain and pain sensitization in a cohort study of people with hand OA.

**Design:**

We examined whether comorbidity burden and individual comorbidities based on the self-administered Comorbidity Index (range: 0–42) at baseline were associated with pain outcomes at baseline and 3 years follow-up. Pain outcomes included hand and overall bodily pain (range: 0–10) as well as pressure pain thresholds at the tibialis anterior muscle (kg/cm^2^) and temporal summation (distal radioulnar joint) as measures of central pain sensitization. We performed linear regression analyses adjusted for age, sex, body mass index, physical exercise and education.

**Results:**

We included 300 and 196 participants in cross-sectional and longitudinal analyses, respectively. Using baseline data, the burden of comorbidities was associated with greater pain in hands (beta ​= ​0.61, 95% CI 0.37, 0.85) and overall body (beta ​= ​0.60, 95% CI 0.37, 0.87). Similar strength of associations was found between comorbidity burden (baseline) and follow-up pain. Among the individual comorbidities, back pain and depression were associated with nearly one unit higher pain score in hands and overall body at both baseline and follow-up. Only back pain was related to lower pressure pain thresholds at follow up (beta ​= ​−0.24, 95% CI −0.50, −0.001).

**Conclusion:**

People with hand OA and greater comorbidity burden, co-existing back pain or depression reported greater pain severity than their counterparts, also 3 years later. These results acknowledge the relevance of accounting for comorbidities in the pain experience in people with hand OA.

## 1 Introduction

The contributors of hand osteoarthritis (OA) pain remain poorly understood, although pain is a frequent symptom and the main reason people approach health services [1,2]. People with OA represent a clinically diverse population and a variety of factors has been found to be associated with OA pain, including factors beyond changes in the joint.

OA pain has traditionally been viewed as a result of joint pathology. In people with hand OA, there is however a discrepancy between the extent of joint damage in the hand and the level of overall hand pain severity [[3], [4], [5]], implying that mechanisms beyond the joint are contributing to the pain experience. Nociplastic pain, defined as pain that arises from altered nociception despite neither tissue nor sensory damage or changes [6], has been acknowledged as a contributor to the OA pain experience [7,8]. Nociceptive pain caused by tissue or sensory damage or changes and nociplastic pain may overlap in chronic painful diseases such as OA [9].

In 1977, George Engel presented a biopsychosocial model to assess pain which incorporates biological, social, psychological, and behavioral domains [10]. A considerable proportion of people with OA present with additional comorbidities which may be associated with pain [11], possibly through elevated pain sensitivity. The European Alliance of Associations for Rheumatology, previously European League Against Rheumatism (EULAR), has highlighted the importance of considering comorbidities in management of hand OA in order to individualize the treatment [12]. A systematic review reported a relation between depression and hand OA pain [13], while conflicting results have been shown between diabetes and hand pain in previous cross-sectional hand OA studies [14,15]. Longitudinal studies of people with knee and hip OA have shown that those with several comorbidities are more likely to experience a worsening of their joint pain [13,16,17].

There is a lack of studies focusing on hand OA and their pain experience, compared to other joints with OA such as knee/hip considering their distinction as non-weightbearing vs. weightbearing. Few previous studies have assessed the longitudinal relationships between comorbidities and pain in people with hand OA [18,19], and they have mainly focused on a few distinct comorbidities. Nor has the comorbidity burden with regard to pain in hand OA been examined. Furthermore, the relationships between comorbidities and pain sensitization have not previously been investigated. There is a need for longitudinal studies to better understand whether comorbidities, and which comorbidities, affect the progression or maintenance of pain in people with hand OA. Thus, the objective of this study was to examine the cross-sectional and longitudinal associations of comorbidities and comorbidity burden to pain and pain sensitization in a longitudinal study of persons with hand OA.

## 2 Methods

### 2.1 Study design and participants

The Nor-Hand study is a hospital-based observational hand OA cohort study, as described in previously published protocols [20,21]. The present longitudinal analyses are based on the baseline data collected in 2016–2017 and the follow-up examination in 2019–2021. Participants were recruited consecutively from the Rheumatology outpatient clinic at Diakonhjemmet Hospital. Detailed inclusion and exclusion criteria have been described in the protocol papers. In short, men and women between 40 and 70 years with hand OA in at least one finger or thumb base joint confirmed by ultrasound and/or clinical examination were included. People with rheumatoid arthritis, spondylarthritis, psoriatic arthritis, psoriasis or hemochromatosis were excluded. Prior to the follow-up examination, all participants were asked about development of systemic inflammatory joint diseases and skin psoriasis and excluded in case of development of such diseases. The study was approved by the Norwegian Regional Committee for Medical and Health Research Ethics (Ref. no: 2014/2057 and 2019/363) and registered at https://clinicaltrials.gov (Ref. no: NCT03083548). The participants received oral and written information about the study and provided their informed consent. They were informed that they could withdraw at any time throughout the study. A user representative was involved during study planning and throughout the study period, who contributed with input on the study design, and helped to interpret and disseminate results to patients.

### 2.2 Specific comorbidities and the total burden of comorbidities

The participants self-reported their comorbidities at baseline using a Comorbidity index [22], which was modified to include 11 pre-defined medical conditions (heart disease, high blood pressure, lung disease, diabetes, ulcer/stomach disease, kidney disease, liver disease, anemia/other blood diseases, cancer, depression and back pain) and three optional conditions. The comorbidity “OA” was removed from the original questionnaire, as all participants had hand OA and it could not be distinguished whether the question was answered according to OA in other joints. The participants were assigned a maximum of three points for each medical condition, including one point for the presence of the problem, one point if receiving treatment for it, and one point if the comorbidity caused activity limitations. The Comorbidity Index sum score was calculated (range: 0–42) to create a measure of total comorbidity burden.

#### 2.2.1 Self-reported pain severity

Measures of pain severity were acquired at baseline and at the 3.5 year follow-up. Pain severity was self-reported according to the Numeric Rating Scale (NRS, range: 0–10) for hand pain and overall bodily pain during the last 24 ​h. Change in pain between baseline and follow-up was calculated for both pain measures. The Patient Acceptable Symptom State (PASS) threshold was defined as a NRS pain score ≥4 [23].

#### 2.2.2 Quantitative sensory testing

To assess central pain sensitization at baseline two trained medical students performed quantitative sensory testing including pressure pain thresholds (PPTs) (kg/cm^2^) at the tibialis anterior and temporal summation (TS) at the left distal radioulnar joint, as described in detail in the protocol [21]. The testing was conducted based on the same predefined protocol throughout the data collection period. To assess the inter-observer reliability nine randomly selected participants were examined by both examiners the same afternoon at baseline.

### 2.3 Covariates

Age, sex, body mass index (BMI), physical exercise and education at baseline were included as potential confounders in linear regression analyses. Information about age and sex was collected from medical records. Height (without shoes) and weight (in light clothing) was measured by medical students and BMI was calculated (kg/m^2^). Physical exercise was assessed by one question: “How many times a week do you exercise for at least 30 ​min?” with four response alternatives ranging from “not regularly” to “3 times a week or more”. The highest level of completed education was reported with seven response alternatives ranging from “7 years elementary school or shorter” to “at least 4 years of university or higher education”.

### 2.4 Statistical analyses

Characteristics of the study population are presented as proportions, mean with standard deviation (SD) or median with interquartile range (IQR), as appropriate. To analyze the pain sensitization variables as continuous variables, but still account for the sex differences, we sex-standardized the PPT tibialis anterior and TS variables by subtracting the mean value from the observed value for each participant. This value was then divided by the SD. Due to differences in pain sensitization between men and women, mean values and SDs were calculated for each sex separately. A value of 0 in a participant corresponds to the mean value in that specific sex, while a value of −1 and 1 corresponds to a value that is one SD below and above the mean value, respectively.

All participants with at least one available pain outcome were included in the cross-sectional analyses. In the longitudinal analyses, participants with missing pain outcome at either baseline or follow-up were excluded. Missing values for covariates received estimated mean values as simple imputations.

The cross-sectional association of absence/presence of the 11 individual comorbidities and the Comorbidity Index sum score (range: 0–42) (explanatory variables) with NRS hand pain, NRS overall bodily pain, PPT and TS (outcome variables) were analyzed using separate linear regression models. The beta values were presented per SD increase of the Comorbidity index. Longitudinal analyses were conducted similarly, using NRS hand pain, NRS overall bodily pain, PPT and TS at follow-up as outcome variables. Analyses were repeated using change in NRS hand and overall bodily pain severity as outcomes. Differences in NRS hand and overall bodily pain equal or above PASS in persons with vs. without the individual comorbidities were assessed by Chi Square test.

For the cross-sectional analysis, we conducted a sensitivity analysis including participants who attended both the baseline and the follow-up examinations. All analyses were adjusted for age, sex, BMI, physical exercise, and education. We also conducted sensitivity analyses where we in addition included time to follow-up (mean 3.5 years) as a confounder. Significance level was set to *p* ​< ​0.05. Statistical analyses were performed with Stata/IC 16.1.

## 3 Results

### 3.1 Participant characteristics

All 300 participants in the baseline examination of the Nor-Hand study were included in the cross-sectional analyses. After a mean follow-up of 3.5 years (range: 2.4–4.2 years), 87 persons were lost to follow-up due to unwillingness to participate (*n* ​= ​56), not possible to get in contact with (*n* ​= ​27) and development of systemic inflammatory joint diseases and/or psoriasis (*n* ​= ​4). Among the 213 participants who attended both examinations, 17 were excluded from the longitudinal analyses due to missing data at either baseline or follow-up, leaving 196 eligible for longitudinal analyses.

Baseline characteristics are listed in Table 1. Overall, the participants in the cross-sectional analyses (*n* ​= ​300) and the participants in the longitudinal analyses (*n* ​= ​196) had similar characteristics. The participants lost to follow-up displayed similar baseline characteristics across demographics, pain and pain sensitization, as the participants included in the cross-sectional and longitudinal analyses (data not shown). The median age among the 300 participants was 61 years and most of the participants were women (89%). The majority presented with two or more comorbidities (77%). The most frequently reported comorbidities were back pain (61%), hypertension (31%), stomach ulcer or other abdominal disease (22%) and depression (16%). Among the participants who reported that they received treatment for hypertension and depression, 79% and 87% reported use of antihypertensives or antidepressants in their list of medications, respectively. Patient-reported antihypertensives or antidepressants are specified in Supplementary Table 4. Self-reported level of pain in the hands and overall body were of similar magnitude, although a large variety was found (Table 1). The majority demonstrated small changes in pain during follow-up with a median (IQR) change of 0 (−1, 1) and 0 (−1, 2) for hand pain and overall bodily pain, respectively.

**Table 1:** Study population characteristics at baseline.

	Cross sectional study *N* ​= ​300	Longitudinal study *N* ​= ​196
Sex, *n* (%) women	266 (89)	168 (86)
Age, median (IQR) years	61 (57–66)	60 (57–66)
Body mass index, mean (SD) kg/m^2^	26.5 (5.0)	26.7 (4.8)
Fulfil ACR hand criteria, *n* (%)	278 (93)	188 (96)
Kellgren Lawrence sum score, median (IQR) [0–128]	28 (15–43)	28 (17–45)
Exercise (≥1 times a week), *n* (%)^a^^,^^b^	204 (69)	137 (70)
Education, n (%) with >1 year of college/university^a^^,^^b^	173 (58)	117 (60)
Comorbidity index sum score, median (IQR) [0−42]	7 (5–11)	5 (3–8)
*Individual comorbidities, n (%):*		
Back pain	184 (61)	122 (62)
Hypertension	92 (31)	59 (30)
Stomach ulcer/other abdominal disease	67 (22)	48 (25)
Depression	49 (16)	33 (17)
Lung disease	41 (14)	26 (13)
Heart disease	32 (11)	23 (12)
Anemia/other blood disease	27 (9)	21 (11)
Diabetes	17 (6)	13 (7)
Cancer	17 (6)	8 (4)
Liver disease	7 (2)	7 (4)
Kidney disease	7 (2)	3 (2)
Temporal summation^c^	1 (0–2)	1 (0–2)
Pressure Pain threshold, mean (SD) kg/cm^2^		
Tibialis anterior muscle^b^	5.5 (2.5)	5.4 (2.6)
NRS hand pain, mean (SD) [0−10]^b^	3.8 (2.3)	3.7 (2.2)
NRS overall bodily pain, mean (SD) [0−10]^b^	4.1 (2.3)	4.0 (2.3)

IQR ​= ​Interquartile Range; SD ​= ​Standard Deviation; ACR ​= ​American College of Rheumatology; NRS ​= ​Numeric Rating Scale. Brackets present possible ranges.

a Data missing for study population *N* ​= ​196: *N* ​= ​1 missing for Exercise and Education.

b Data missing for study population *N* ​= ​300: *N* ​= ​2 missing for Education, NRS hand pain and Temporal summation; *N* ​= ​4 missing for NRS overall bodily pain; *N* ​= ​6 for Exercise and *N* ​= ​9 for PPT tibialis anterior.

c Temporal Summation represents change in pain on NRS from first tap to 5th or 10th tap.

### 3.2 Comorbidities and self-reported pain severity

The Comorbidity Index at baseline was significantly associated with greater pain severity in hands and overall body at baseline (Table 2) and follow-up (Table 3). The results remained similar in the sensitivity analyses, were we additionally adjusted for the time to follow-up. An increase of the Comorbidity Index corresponding to its SD (3.8 points on the 0–42 scale) was associated with 0.61 (95% CI 0.37, 0.85) and 0.48 (95% CI 0.19, 0.84) unit higher hand pain score at baseline and follow-up, respectively. Similar results were found for overall bodily pain (Table 2, Table 3).

**Table 2:** Associations between comorbidities at baseline and pain severity at baseline.

	Hand pain severity (NRS 0–10) Beta (95% CI)	Overall bodily pain severity (NRS 0–10) Beta (95% CI)
Comorbidity index sum score^a^	**0.61 (0.37, 0.85)**	**0.60 (0.37, 0.87)**
Individual comorbidities		
Back pain	**0.98 (0.49, 1.48)**	**1.07 (0.56, 1.58)**
Hypertension	**0.76 (0.18, 1.33)**	0.39 (−0.22, 0.99)
Stomach ulcer/other abdominal disease	0.60 (−0.08, 1.16)	0.51 (−0.11, 1.14)
Depression	**0.99 (0.35, 1.64)**	**0.75 (0.07, 1.42)**
Lung disease	0.26 (−0.45, 0.97)	0.08 (−0.66, 0.81)
Heart disease	0.69 (−0.10, 1.48)	0.64 (−0.18, 1.45)
Anemia/other blood disease	−0.02 (−0.88, 0.85)	−0.20 (−1.09, 0.69)
Diabetes	0.09 (−0.98, 1.17)	−0.43 (−1.54, 0.68)
Cancer	1.00 (−0.05, 2.05)	0.69 (−0.40, 1.78)
Liver disease	1.23 (−0.38, 2.85)	1.18 (−0.48, 2.85)
Kidney disease	0.87 (−0.75, 2.48)	−0.64 (−2.31, 1.03)

NRS ​= ​Numeric Rating Scale; CI ​= ​confidence interval.

∗∗Adjusted for age, sex, BMI, physical exercise and education; **Bold** indicates statistically significant associations.

a Beta values (95% CI) per one SD (3.8).

**Table 3:** Associations between comorbidities at baseline and pain severity at the 3.5 year follow-up.

	Hand pain severity (NRS 0–10) Beta (95% CI)	Overall bodily pain severity (NRS 0–10) Beta (95% CI)
Comorbidity index sum score^a^	**0.48 (0.19, 0.84)**	**0.57 (0.25, 0.89)**
Individual comorbidities		
Back pain	**0.67 (0.07, 1.28)**	**0.89 (0.27, 1.51)**
Hypertension	−0.02 (−0.74, 0.70)	−0.26 (−1.00, 0.49)
Stomach ulcer/other abdominal disease	0.58 (−0.16, 1.31)	0.74 (−0.01, 1.50)
Depression	**1.01 (0.22, 1.79)**	**0.97 (0.16, 1.78)**
Lung disease	0.28 (−0.59, 1.15)	−0.03 (−0.94, 0.87)
Heart disease	**1.29 (0.38, 2.19)**	**1.08 (0.14, 2.01)**
Anemia/other blood disease	0.20 (−0.77, 1.17)	−0.23 (−1.23, 0.77)
Diabetes	−0.23 (−1.54, 1.09)	0.001 (−1.37, 1.37)
Cancer	0.11 (−1.38, 1.60)	0.15 (−1.39, 1.69)
Liver disease	1.11 (−0.47, 2.70)	0.34 (−1.30, 1.99)
Kidney disease	−0.12 (−2.55, 2.29)	0.12 (−2.39, 2.61)

NRS ​= ​Numeric Rating Scale; CI ​= ​confidence interval.

∗∗Adjusted for age, sex, BMI, physical exercise and education; **Bold** indicates statistically significant associations.

a Beta values (95% CI) per one SD (3.8).

Among the individual comorbidities, the associations were less consistent (Table 2, Table 3). Having back pain or depression were significantly associated with nearly one unit higher hand pain or overall bodily pain at both baseline and follow-up. Hypertension was associated with hand pain at baseline only. Heart disease was associated with both pain outcomes, but was only statistically significant at follow-up. The sensitivity analysis also displayed a significant association between heart disease and hand pain (Supplementary Table 1).

**Fig. 1:**
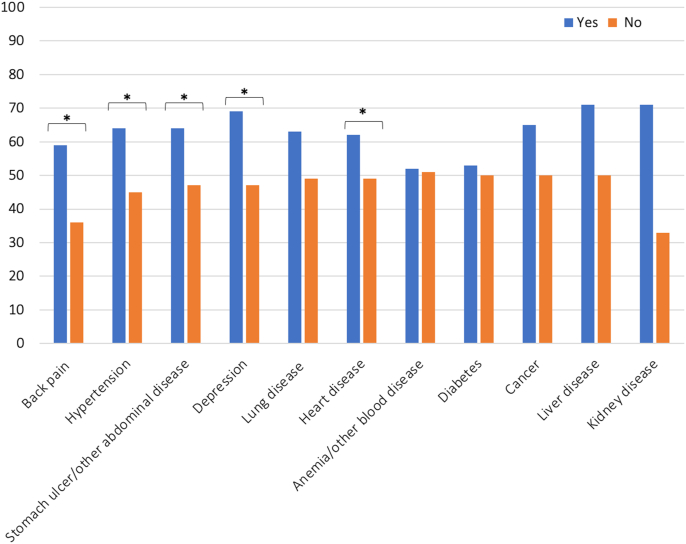
Proportion (%) of participants with NRS hand pain ≥4 (PASS) at baseline, by comorbidity. ∗Significant difference between groups by comorbidity (yes/no), *p* ​< ​0.05. NRS ​= ​Numerical Rating Scale; PASS ​= ​Patient Acceptable Symptom State.

A total of 49% and 54% had NRS hand pain and overall bodily pain of 4 or more, representing the PASS threshold, respectively [23]. The proportions of participants with hand pain equal or above PASS are shown in Fig. 1. Pain above PASS was statistically significantly more common in participants with back pain, hypertension, stomach ulcer/other abdominal disease, and depression than in participants without these comorbidities. Large differences in the proportions with pain above PASS were also found for participants with vs. without cancer, liver disease and kidney disease, although the differences were not statistically significant (Fig. 1). Similar results were found for NRS overall bodily pain (data not shown).

No associations were found between the Comorbidity Index and changes in pain severity from baseline to follow-up in the hands (beta ​= ​0.02, 95% CI −0.07, 0.11) or overall body (beta ​= ​−0.01, 95% CI −0.10, 0.09). Similarly, we observed no significant associations between individual comorbidities and changes in pain severity (data not shown).

### 3.3 Comorbidities and QST measures of pain sensitization

A weak but statistically significant association between the Comorbidity Index and TS at baseline was observed. Back pain at baseline was associated with lower PPT at tibialis anterior at follow-up only. Otherwise, no statistically significant associations were found (Supplementary Tables 2 and 3). The inter-observer reliability was moderate with intraclass correlation coefficients (two-way mixed-effects model, absolute agreement, individual measure) of 0.43 for PPT at the tibialis anterior muscle and 0.56 for TS. The analyses were repeated with only the participants that were assessed by the examiner that performed most assessments which yielded similar results as in our main analyses.

## 4 Discussion

The Nor-Hand study is the first to explore the longitudinal relationships of the comorbidity burden and individual comorbidities with pain severity and pain sensitization in a large hand OA population. In this cohort, greater comorbidity burden, back pain and depression at baseline contributed to worse pain severity in hands and overall body at baseline and predicted worse pain outcomes at 3 years follow-up. Other individual comorbidities, such as hypertension and heart disease, showed significant associations with some pain outcomes, but the associations were not consistent. The associations between comorbidities and measures of central sensitization were mostly non-significant, suggesting that the observed associations to pain severity in most cases could not be explained by hypersensitivity to pain.

The Nor-Hand study is unique by having longitudinal data. Our results suggest that comorbidity burden, back pain and depression contribute to sustained pain in people with hand OA. On the other hand, the comorbidities did not affect the change in pain from baseline to follow-up, suggesting that pain progression or fluctuations in pain are driven by other factors. Contrary to our study, a systematic review found that a greater comorbidity count was associated with worsening of knee and hip pain severity [24]. Most of our study participants demonstrated small changes in pain, which may explain the lack of association between comorbidity burden and change in pain. Furthermore, since people with hand OA may experience flares of the disease, their pain can fluctuate from day to day, and it may thus be challenging to capture the long-term change of pain with only two time points. Our results on the associations between the burden of comorbidities and hand pain severity are in line with a previous hand OA study [14], which demonstrated that the number of comorbidities were associated with disease burden in terms of hand pain and function assessed by the Australian/Canadian Osteoarthritis Hand Index (AUSCAN) [25]. Our findings of NRS hand pain and overall bodily pain with beta values of 0.48–0.61 per one SD (3.8 points) increase of the Comorbidity Index, suggest that a difference of 6–8 points on the Comorbidity Index would be clinically relevant [26]. This corresponds to the mean value in this population.

The most frequently reported comorbidities were back pain, hypertension, stomach ulcer/other abdominal disease and depression. Presence of back pain and depression showed consistent associations with greater pain severity in the hands and overall body, and the observed associations were clinically relevant with beta values of around 1 point on the NRS [26]. These results are in line with previous OA studies. Associations between back pain and knee pain severity were found in a previous study of people with symptomatic knee OA [27]. Likewise, a hospital-based hand OA study found that participants with self-reported depression and/or anxiety reported higher levels of hand pain severity than participants without such symptoms [28]. Using data from the same cohort, we have also previously shown that symptoms of depression and anxiety using the Hospital Depression and Anxiety scale were related to greater pain severity [29]. The observed association in this study was numerically weaker than the results from the previous analyses, which may be due to different assessment of depression (yes/no vs. burden of symptoms).

The relationships of hypertension and heart disease with pain severity were not consistent across our two pain outcomes and two time points, and our results on these analyses should therefore be treated with caution. Conflicting results regarding hypertension and pain have been found in previous hand OA studies [18,30]. Previous studies have shown associations between heart disease and symptomatic hand OA, which remained after accounting for possible shared risk factors such as age, sex and BMI [18,31]. Other potential shared risk factors include low-grade inflammation [18]. Importantly, pain may also contribute to the development of hypertension or heart disease through inactivity, and cardiovascular diseases may be side effects of analgesic treatment with non-steroidal anti-inflammatory drugs (NSAIDs) [[32], [33], [34]]. In these analyses, we did not have historical data on the use of NSAIDs and were thus not able to adequately adjust for medication in our analyses.

Although we did not find significant relations for the remaining comorbidities, we did observe large differences in pain between participants with cancer, liver and kidney disease when compared to participants without these comorbidities. The small size of the groups with cancer, liver and kidney disease in this study, and thus a low statistical power, likely impacted the ability to detect any significant differences. Pain is a common consequence of cancer [35]. Both chronic pain and cancer therapy may lead to central sensitization and thus increased overall bodily pain [36], as shown in our study. Paracetamol and NSAIDs are commonly used analgesics for OA [37]. Long-term use of NSAIDs is assumed to partly explain the documented association between gastrointestinal diseases and symptoms and quality of life in OA [32,38]. Paracetamol is found to increase gastrointestinal and hepatic adverse events, and renal dysfunctions have been ascribed to use of NSAIDs [37]. The observed differences in pain in this study for these groups may be attributed to possible side-effects of long-term use of paracetamol and NSAIDs due to OA-related pain.

The underlying mechanisms linking comorbidities to pain in the hands and overall body remains unknown. Based on previous literature [8,39,40], altered pain physiology and mechanisms of central sensitization could theoretically explain the observed associations between comorbidities and self-reported pain severity. However, comorbidity burden, back pain and depression showed mostly non-significant associations with measures of central sensitization, questioning the importance of this mechanism behind the observed associations in this study. Having the moderate reliability of quantitative sensory testing in mind, further investigation of this topic is warranted in future studies. The spine is frequently affected by OA [41], and the observed association between back pain, hand pain and overall bodily pain may thus be explained by generalized OA leading to pain at several body sites. Other examples of shared risk factors for back pain and pain at other sites, as well as for depression and pain, include genetic factors, life style factors, cognitive functioning such as self-efficacy, previous experiences and social support [42], but further exploration of these factors were beyond the scope of the current work. No previous studies have explored whether treating comorbidities leads to less pain in a hand OA population. The shared risk factors that are listed above may be relevant treatment targets, for example through cognitive behavioral therapy or lifestyle modifications.

Our study is strengthened by the inclusion of a study sample with wide range of symptoms. The participants were not required to fulfill the ACR hand OA criteria or a certain level of pain before enrollment, which may have increased the generalizability of our results to people with less symptomatic hand OA. The following limitations should be addressed: Due to the explorative nature of this study, the results should be validated in other cohorts. There was a 27% loss to follow-up in this study. The older population included in our study in combination with the outbreak of the corona pandemic in 2020 during the data collection, likely impacted the willingness to participate further in the study. However, this group displayed similar characteristics as the participants included in the analyses. The Comorbidity Index was self-reported, and the participants may not accurately report their concurrent conditions. The strength of the association between back pain and overall bodily pain may have been influenced by the level of back pain experienced by the participants and should be interpreted accordingly. The participants were recruited from secondary care and may have more pain than people with hand OA in primary care, influencing the generalizability. An overrepresentation of people with higher education, good physical and mental health and a high proportion of women may have biased the results. The inter-reader reliability of quantitative sensory testing was lower than in previous studies [42,43], which may have diluted the strength of the association. Considering the similar results in the repeated analyses which only included the participants examined by the examiner which performed the majority of the assessments, it indicates the measures are not influenced by the moderate inter-observer reliability.

In summary, a higher burden of comorbidity, presence of back pain and depression were associated with greater pain severity and long-term pain, but not with change in pain. These findings underscore the relevance of taking comorbidities into account in the management of people with hand OA, as well as the multifactorial nature of the hand OA pain experience.

## Author contributions

Substantial contributions to the conception and design of the study: EM, TN, HD, PSP, TKK and IKH, or acquisition/analysis of data: EM, PSP, MG, KM, and IKH. Interpretation of data and drafting of the manuscript or revising it critically: EM, TN, HD, HBH, PSP, MG, KM, TKK and IKH. Final approval of the publication: EM, TN, HD, HBH, PSP, MG, KM, TKK and IKH.

## Role of the funding source

The Nor-Hand study was funded by the Norwegian Research Council (project number: 328657), ADVANCE grant from 10.13039/100004319Pfizer/10.13039/501100022186Lily, South-East Norway Regional Health Authority, Pahles foundation, Simon Fougner Hartmanns Family foundation and Trygve Gythfeldt’s research foundation. Dr. Neogi was supported by 10.13039/100000002NIH K24 AR070892, P30 AR072571 and R01 AR062506. The funders were not involved in the study design, collection, analysis or interpretation of data.

## Declaration of competing interest

TKK reports grants and personal fees from 10.13039/100006483AbbVie, 10.13039/100004336Novartis and 10.13039/100004319Pfizer, grants from BMS and UCB, personal fees from; personal fees from AbbVie, Amgen, Biogen, Celltrion, Egis, Eli Lilly, Evapharma, Ewopharma, Gilead, Hikma, Mylan, Oktal, Sandoz and Sanofi and participation in AbbVie Advisory Board outside the submitted work. HBH reports personal fees from AbbVie, Novartis, Lilly and UCB, as well as participation in AbbVie Advisory Board outside the submitted work. IKH reports grants from 10.13039/100004319Pfizer/Lilly (ADVANCE), personal fees from Novartis and GSK, and a leadership/fiduciary role related to OARSI (unpaid) outside the submitted work. EM, HD, PSP, MG and KM have nothing to disclose.
