# Supplementary material for: Comorbidities in people with hand OA and their associations with pain severity and sensitization: Data from the longitudinal Nor-Hand study
Source: Osteoarthr Cartil Open. 2023 May 5;5(3):100367. doi: 10.1016/j.ocarto.2023.100367 (PMC10206186; doi:10.1016/j.ocarto.2023.100367)
Supplement: Multimedia component 1 [file mmc1.docx]

**Supplementary material:**

| **Supplementary table 1:**  Associations between comorbidity at baseline and NRS pain severity at baseline.  (N=193, who attended both baseline and follow-up examinations) | | |
| --- | --- | --- |
|  | **Hand pain**  **severity**  (0-10)  Beta (95% CI) | **Overall bodily pain severity**  (0-10)  Beta (95% CI) |
| Comorbidity burden* | **0.57 (0.23, 0.87)** | **0.57 (0.23, 0.91)** |
| Back pain | **0.59 (0.29, 0.90)** | **0.70 (0.39, 1.01)** |
| Hypertension | 0.31 (-0.07, 0.67) | 0.29 (-0.10, 0.68) |
| Stomach ulcer/other abdominal disease | 0.18 (-0.13, 0.48) | 0.09 (-0.22, 0.41) |
| Depression | 0.29 (-0.03, 0.61) | 0.22 (-0.11, 0.56) |
| Lung disease | 0.04 (-0.23, 0.30) | 0.04 (-0.24, 0.32) |
| Heart disease | **0.34 (0.06, 0.62)** | 0.25 (-0.05, 0.54) |
| Anemia/other blood disease | -0.002 (-0.32, 0.19) | -0.17 (-0.45, 0.17) |
| Diabetes | -0.10 (-0.51, 0.30) | -0.24 (-0.67, 0.18) |
| Cancer | 0.19 (-0.12, 0.49) | 0.16 (-0.15, 0.48) |
| Liver disease | 0.26 (-0.06, 0.59) | 0.23 (-0.11, 0.57) |
| Kidney disease | 0.10 (-0.20, 0.40) | 0.10 (-0.21, 0.41) |
| NRS=Numeric Rating Scale; CI=confidence interval.  *Beta values (95% CI) per one SD (3.8).  **Adjusted for age, sex, BMI, physical exercise and education; **Bold** indicates statistically significant associations. | | |

| **Supplementary table 2:**  Associations between comorbidity at baseline and pain sensitization at baseline. | | |
| --- | --- | --- |
|  | **TS**  Beta (95% CI) | **PPT Tibialis Anterior**  Beta (95% CI) |
| Comorbidity burden* | **0.21 (0.07, 0.35)** | -0.07 (-0.17, 0.03) |
| Back pain | 0.20 (-0.10, 0.49) | 0.10 (-0.10, 0.30) |
| Hypertension | 0.21 (-0.12, 0.56) | -0.08 (-0.31, 0.16) |
| Stomach ulcer/other abdominal disease | 0.23 (-0.12, 0.58) | 0.12 (-0.37, 0.12) |
| Depression | 0.18 (-0.20, 0.57) | -0.15 (-0.41, 0.12) |
| Lung disease | -0.34 (-0.07, 0.76) | 0.03 (-0.26, 0.32) |
| Heart disease | 0.37 (-0.09, 0.84) | -0.13 (-0.45, 0.20) |
| Anemia/other blood disease | -0.10 (-0.60, 0.41) | 0.05 (-0.30, 0.40) |
| Diabetes | 0.02 (-0.65, 0.61) | -0.40 (-0.83, 0.04) |
| Cancer | 0.27 (-0.34, 0.89) | 0.15 (-0.27, 0.58) |
| Liver disease | 0.18 (-0.77, 1.12) | -0.52 (-1.17, 0.14) |
| Kidney disease | 0.59 (-0.35, 1.53) | 0.05 (-0.70, 0.61) |
| PPT=Pressure detection Pain Threshold; TS=Temporal Summation; CI=confidence interval.  *Beta values (95% CI) per one SD (3.8).  **Adjusted for age, sex, BMI, physical exercise and education; **Bold** indicates statistically significant associations. | | |

| **Supplementary table 3:**  Associations between comorbidity at baseline and pain sensitization at the 3.5 year follow-up. | | |
| --- | --- | --- |
|  | **TS**  Beta (95% CI) | **PPT Tibialis Anterior**  Beta (95% CI) |
| Comorbidity burden* | 0.01 (-0.16, 0.17) | -0.09 (-0.23, 0.04) |
| Back pain | 0.22 (-0.10, 0.53) | **-0.24 (-0.50, -0.001)** |
| Hypertension | -0.0004 (-0.3, 0.37) | 0.05 (-0.25, 0.34) |
| Stomach ulcer/other abdominal disease | 0.21 (-0.17, 0.59) | 0.14 (-0.16, 0.44) |
| Depression | -0.16 (-0.57, 0.25) | 0.06 (-0.10, 0.21) |
| Lung disease | -0.05 (-0.50, 0.40) | -0.04 (-0.40, 0.31) |
| Heart disease | -0.36 (-0.83, 0.11) | -0.04 (-0.41, 0.33) |
| Anemia/other blood disease | -0.24 (-0.26, 0.74) | -0.09 (-0.48, 0.31) |
| Diabetes | 0.24 (-0.39, 0.87) | 0.06 (-0.45, 0.56) |
| Cancer | -0.44 (-1.21, 0.32) | -0.36 (-0.98, 0.24) |
| Liver disease | 0.48 (-0.32, 1.29) | -0.35 (-1.00, 0.30) |
| Kidney disease | -0.03 (-1.28, 1.22) | 0.32 (-0.67, 1.31) |
| PPT=Pressure detection Pain Threshold; TS=Temporal Summation; CI=confidence interval.  *Beta values (95% CI) per one SD (3.8).  **Adjusted for age, sex, BMI, physical exercise and education; **Bold** indicates statistically significant associations. | | |

| **Supplementary table 4:** Patient-reported antihypertensives and antidepressants that were used alone or in combination by participants in the Nor-Hand study. | |
| --- | --- |
| Antihypertensives: | - Thiazides: Hydrochlorothiazide with or without Amiloride, Bendroflumethiazide with Kaliumchloride - Angiotensin II receptor antagonists: Valsartan, Losartan, Candesartan, Irbesartan, Olmesartan - Angiotensin-converting enzyme (ACE) inhibitors: Lisinopril, Ramipril - Beta blockers: Metoprolol, Propanolol, Atenolol - Calcium channel blockers: Verapamil, Lerkanidipin, Amlodipin - Unspecified antihypertensive |
| Antidepressants: | - Tricyclic antidepressants: Amitriptyline - Tetracyclic antidepressants: Mirtazapine - Selective serotonin reuptake inhibitors: Escitalopram, Citalopram - Serotonin and norepinephrine reuptake inhibitors: Venlafaxine - Other antidepressants: Bupropion - Unspecified antidepressant |
